# *Helicobacter pylori* CagA Protein Negatively Regulates Autophagy and Promotes Inflammatory Response via c-Met-PI3K/Akt-mTOR Signaling Pathway

**DOI:** 10.3389/fcimb.2017.00417

**Published:** 2017-09-21

**Authors:** Na Li, Bin Tang, Yin-ping Jia, Pan Zhu, Yuan Zhuang, Yao Fang, Qian Li, Kun Wang, Wei-jun Zhang, Gang Guo, Tong-jian Wang, You-jun Feng, Bin Qiao, Xu-hu Mao, Quan-ming Zou

**Affiliations:** ^1^Department of Clinical Microbiology and Immunology, Southwest Hospital & College of Medical Laboratory Science, Third Military Medical University Chongqing, China; ^2^Department of Microbiology and Biochemical Pharmacy, National Engineering Research Center for Immunobiological Products, College of Pharmacy, Third Military Medical University Chongqing, China; ^3^Institute of Cardiovascular Disease, General Hospital of Jinan Military Region Jinan, China; ^4^Emei Sanatorium of PLA Rocket Force Emeishan, China; ^5^Department of Medical Microbiology and Parasitology, Zhejiang University School of Medicine Hangzhou, China

**Keywords:** *Helicobacter pylori*, autophagy, CagA, c-Met, SQSTM1

## Abstract

Cytotoxin-associated-gene A (CagA) of *Helicobacter pylori* (*H. pylori*) is a virulence factor that plays critical roles in *H. pylori*-induced gastric inflammation. In the present study, gastric biopsies were used for genotyping *cagA* and *vacA* genes, determining the autophagic activity, and the severity of gastric inflammation response. It was revealed that autophagy in gastric mucosal tissues infected with *cagA*^+^ *H. pylori* strains was lower than the levels produced by *cagA*^−^ *H. pylori* strains, accompanied with accumulation of SQSTM1 and decreased LAMP1 expression. *In vitro*, deletion mutant of *cagA* gene resulted in increased autophagic activity, and decreased expression of SQSTM1 and cytokines, whereas over-expression of CagA down-regulated the starvation-induced autophagy, and induced more production of the cytokines. Moreover, the production of the cytokines was increased by inhibition of autophagy, but decreased by enhancement of autophagy. Deletion of CagA decreased the ability to activate Akt kinase at Ser-473 site and increased autophagy. c-Met siRNA significantly affected CagA-mediated autophagy, and decreased the level of p-Akt, p-mTOR, and p-S6. Both c-Met siRNA and MK-2206 could reverse inflammatory response. *H. pylori* CagA protein negatively regulates autophagy and promotes the inflammation in *H. pylori* infection, which is regulated by c-Met-PI3K/Akt-mTOR signaling pathway activation.

## Introduction

*Helicobacter pylori (H. pylori)* is a Gram-negative bacterium causing gastritis, peptic ulcer disease and gastric adenocarcinoma (Suerbaum and Michetti, 2002). Although *H. pylori* could induce strong inflammation, it is not able to clear the bacterium, resulting in persistent infection. Cytotoxin-associated gene A (CagA), one of *H. pylori* virulence factors, is an effector secreted by the type IV secretion system into gastric epithelial cells, and undergoes tyrosine phosphorylation, and activates a series of intracellular signal transduction reactions, resulting in severe tissue inflammation and damage (Gunn et al., 1998). Generally, *H. pylori* strains expressing CagA protein is more virulent, and leading to severe gastritis (Fischer et al., 2001). CagA is able to activate the transcription factor, NF-κB, and translocate it into the nucleus, where it up-regulates transcription of interlukin-8 (IL-8), a chemotactic and inflammatory cytokine (Brandt et al., 2005). However, the specific mechanism that CagA-positive strains induce inflammation remains unclear.

Macroautophagy (hereafter autophagy) has an important role in controlling intracellular environment. The damaged cell organelles, proteins, or invading microorganisms are sequestered into autophagosomes, and finally delivered into autolysosomes for degradation (Shintani and Klionsky, 2004). In the case of infection of pathogenic microorganism, the final consequence of the infection was decided by the evolving struggle between the host cells and invading microbes, and autophagy plays a critical role in the struggle. A number of important pathogens could be degraded by autolysosomes, such as, *Listeria monocytogenes* (Py et al., 2007), group A Streptococcus (Nakagawa et al., 2004), and *Francisella tularensis* (Cremer et al., 2009). However, some pathogenic bacteria also develop some mechanisms to subvert autophagy to survive in cells, eventually leading to the occurrence of various diseases, such as, Shigella (Kayath et al., 2010) and *Mycobacterium tuberculosis* (Shin et al., 2010).

It has been demonstrated that the induction of autophagosome formation or autophagy depends on the vacuolating cytotoxin (VacA), which is another virulent factor of *H. pylori* (Terebiznik et al., 2009). In turn, autophagy can eliminate intracellular *H. pylori* and may decrease the stability of intracellular VacA and ameliorate toxin-mediated cellular vacuolation (Terebiznik et al., 2009), despite the fact that autophagy is not sufficient to block vacuole biogenesis and pathogenesis (Zavros and Rogler, 2012). Recently, Tsugawa et al. showed that intracellular CagA is degraded by autophagy and short lived in AGS cells (Tsugawa et al., 2012), but whether or not autophagy regulated by CagA in *H. pylori*-induced gastric inflammation have never been explored.

Therefore, the purpose of this article was to determine the effect of CagA on autophagy of gastric epithelial cells and the production of autophagy-regulated proinflammatory cytokines in *H. pylori* infection.

## Materials and methods

### Patients and specimens

Consecutive patients who underwent upper endoscopy due to dyspeptic symptoms at Southwest Hospital, Chongqing, China during January 2013 and December 2014, were recruited. One hundred and six (49 women and 57 men with age of 43 ± 20 years) patients were eligible for enrollment into the *H. pylori* positive group if they had a positive [^13^C] urea breath test, a positive rapid urease test, and *H. pylori* culture. Eleven (six women and five men with age of 35 ± 20 years) with normal gastric mucosa were eligible for enrollment into the *H. pylori* negative group, and the clinical characteristics are shown in Supplementary Table 2. The study was approved by the Institutional Review Board at Third Military Medical University, and all patients signed informed consent before participation. All experiments were performed in accordance with relevant guidelines and regulations.

*H. pylori* was successfully isolated from 106 patients, and genotyping for *cagA* and *vacA* was performed for 106 isolates. All *H. pylori* strains carry the *vacA* gene. To exclude the effect of VacA, the toxigenic vacA genotype (*vacA*^*s1m1*^, 42 cases), expressing a functional VacA toxic, were excluded from the study. The rest of the cases include: normal control (11 cases), *cagA*^−^/*vacA*^*s1m2*^ (7 cases), *cagA*^+^/*vacA*^*s1m2*^ (57 cases). To ensure that approximately equal numbers of each group, 23 selective patients were chosen randomly for analyzing autophagy and inflammation, dividing into normal control (8 cases), *cagA*^−^/*vacA*^*s1m2*^ (7 cases), *cagA*^+^/*vacA*^*s1m2*^ (8 cases).

### Evaluation of inflammation score for gastric biopsy samples

The selected gastric biopsy samples among the genotype subgroups were obtained to perform H&E staining. The intensity of inflammation was evaluated independently by two pathologists according to previously established criteria. The degree of neutrophil infiltration, mononuclear cell infiltration, atrophy, and metaplasia was assessed according to the updated Sydney classification as follows: 0, absent; 1, minimal; 2, mild; 3, moderate; 4, marked. So the biopsies were from different stages of gastritis (Dixon et al., 1996).

### Genotyping for cagA and vacA genes

*H. pylori* infection status was detected by rapid urease test, bacterial culture, ^13^C-urea breath test, and histological examination (Vaira et al., 1999). In patients with positive culture, *H. pylori* isolates were subcultured for a maximum of five passages, and genomic DNA was extracted to genotype for the *cagA* and *vacA* genes, as previously described (Argent et al., 2008). The primers used for PCR amplification and nucleotide sequencing are listed in Supplementary Table 1.

### Cell line and *H. pylori* strains

AGS (a human gastric cancer cell line) purchased from the cell bank of Chinese Academy of Sciences, were cultured in F12 cell culture medium (Gibco, Grand Island, NY, USA, #11765-054) supplemented with 10% FBS (Gibco, #10099-141) in a humidified incubator (5% CO_2_) at 37°C. The starvation condition was established by culturing the cells with serum-free medium for 4 h.

The wide-type *cagA*^+^*/vacA*^+^ *H. pylori* strain, NCTC11637 (*Hp-*WT, obtained from ATCC), *cagA*-knockout *H. pylori* with NCTC11637 background (*Hp*-Δ*cagA*, kindly provided by Dr. Sasakawa (Asahi et al., 2000; Suzuki et al., 2009) and *H. pylori cagA*-knockout complementation mutant (*Hp*-*c-cagA*, constructed by our group), were cultured on brain-heart infusion medium (10% rabbit blood) under microaerophilic conditions (5% O_2_, 10% CO_2_, and 85% N_2_) at 37°C. *Hp*-*c-cagA* mutant was obtained by amplifying the cDNA fragments of *cagA* gene from the gene of NCTC11637 by polymerase chain reaction (PCR), and the primers of PCR is following: forward: 5′-GCGCTCGAGATGACTAACGAACC-3′; reverse: 5′-GCGCTGCAGTTAAGATTTTTGG-3′. The product of PCR was digested with *Xho*I and *Pst*I, and then ligating the cDNA fragments of *cagA* gene between *cagA*-upstream and -downstream sequences cloned on the pHel3 shuttle vector. The pHel3 shuttle vector with *cagA* gene was electroporated into *Hp-*Δ*cagA* cells carrying the kanamycin resistance. *Hp-c-cagA* clones were cultured in brain-heart infusion medium as previous described.

AGS cells transfected with plasmids and/or siRNAs were infected with *Hp*-WT, *Hp*-Δ*cagA*, or *Hp*-*c-cagA*, respectively, with different multiplicity of infection (MOI = 10, 50, 100, 200), for 6 h. Cells without infection served as controls.

### Reagents and antibodies

Rapamycin (Rapa, R8781), 3-methyladenine (3-MA, M9281), bafilomycin A1 (Baf-A1, B1793), antibodies against ATG12 (WH0009140M1), MAP1LC3B (L7543), and ATG5 (WH0009474M1) were purchased from Sigma-Aldrich (Shanghai, CHINA), and MK-2206 2HCL (S1078) purchased from Selleckchem (Houston, TX, USA). Antibodies against Akt (9272), mTOR (2972), AMPK (2532), phospho-Akt (Ser473) (4060), LAMP1 (9091), phospho-mTOR (Ser2448) (5536), phospho-S6 ribosomal protein (Ser235/236) (2211), ribosomal protein (2217), phospho-c-Met (Y1234/Y1235) (4033), and c-Met (4560) were obtained from Cell Signaling Technology (Beverly, MA, USA), whereas antibodies against β-actin (sc-10731), VacA (sc-25790), CagA (sc-17450), phospho-tyrosine (PY99) (sc-7020) and SQSTM1 (sc-28359), and siRNAs specific for SQSTM1 (human, sc-29679), ATG12 (human, sc-72578), c-Met (human, sc-29397), and ATG5 (human, sc-41445), along with a control siRNA (sc-44230) were obtained from Santa Cruz Biotechnology (CA, USA).

### Immunohistochemistry for SQSTM1

Gastric biopsy sections from patients infected with different genotypes of *H. pylori* were stained using SQSTM1 antibody (Enzo Life Sciences, BML-PW9860-0025) by the DAB reagent technique. SQSTM1 staining was assessed by three pathologists with no prior knowledge of the different groups, and scored as 0 (no staining), 1 (< 10% of SQSTM1 staining), 2 (10–50% of SQSTM1 staining), or 3 (>50% of SQSTM1 staining).

### Western blotting, quantitative RT-PCR, NF-κB activity, and ELISA

The protein level of SQSTM1, LAMP1, phospho-mTOR (Ser2448), MAP1LC3B, ATG12, ATG5, AMPK, phospho-Akt (Ser473), Akt, mTOR, ribosomal protein, phospho-c-Met (Y1234/Y1235), c-Met, phospho-S6 ribosomal protein (Ser235/236), VacA, CagA, and phospho-tyrosine (PY99) were performed to determine by Western blotting in gastric tissues or AGS cells as described previously (Tang et al., 2015).

Trizol reagent (Invitrogen, 15596-026) was used to extract total RNA from gastric biopsy sections. qRT-PCR analysis of gastric biopsy sections from patients for the mRNA of *SQSTM1, BECN1, IL-8, TNF-*α, β*-actin*, and *IL-1*β were performed by using PrimeScript RT-PCR kits (Takara, Tokyo, Japan, DRR037), and run on a Bio-Rad IQ5 thermocycler (Bio-Rad Laboratories, Inc., Hercules, USA), β*-actin* as an internal control. The conditions for PCR were as follows: 1 cycle of 95°C for 30 s, 40 cycles of 95°C for 6 s, 60°C for 6 s, and 72°C for 31 s, and the primer sequences used are shown in Supplementary Table 1.

AGS cells were cotransfected with Renilla control vector (*pRL-TK*, Promega, #2241) and luciferase reporter vector *pNF-*κ*B-TA-Luc* (Clontech, #631904) with lipofectamine 2000 (the ratio of 20:1) for 1 day, followed with *H. pylori* infection. The Dual-Luciferase Reporter Assay System (Promega, E1910) detected firefly luciferase activity and Renilla luciferase activity according to the manufacture's protocol.

The supernatant of AGS cells with different treatment were detected by DuoSet ELISA Development System (IL-8, IL-1β, and TNF-α; R&D, Minneapolis, USA) as our previous study (Tang et al., 2016).

### Transfection of AGS with plasmids and/or siRNAs

The *GFP-MAP1LC3B* plasmid and *RFP-MAP1LC3B* expression plasmid were kindly provided by Dr. Tamotsu Yoshimori (Department of Cell Biology, National Institute for Basic Biology, Presto, Japan) and Dr. Maria Colombo (Universidad Nacional de Cuyo, Mendoza, Argentina), respectively. The CagA expression plasmid, *pEGFP-C1*-*CagA* (*GFP-CagA*) (Asahi et al., 2000; Suzuki et al., 2009), was kindly provided by Dr. Chihiro Sasakawa. The *cagA* mutant plasmid, *pEGFP-C1*-*CagA-Mut* (*GFP-CagA-Mut*) was constructed by Life Technologies, Shanghai, China, and a series of CagA mutants with the Tyr residues of 899, 918, and 972 being substituted by Ala were generated from a plasmid-encoding fragment of *cagA* gene of *H. pylori* ATCC 26695 on pBluescript (Promega, Madison, USA) using a Gene Editor *in vitro* Site-Directed Mutagenesis System (Promega). These mutants were at the sites 2,695–2,697, 2,752–2,754, and 2,914–2,915 bp, respectively, started from the sequence ATG. Lipofectamine 2000 (Invitrogen, #11668019) was used to transfect plasmids and/or siRNAs into AGS cells. 3 × 10^6^ AGS cells were seeded into a 100-mm dish and incubated with transfection complexes containing 100 nM siRNA for 24 h.

### Immunoprecipitation assays

AGS cells were harvested in RIPA buffer on crushed ice, and centrifuged at 6,000 rpm for 5 min, and commercial Lowry Assay (Bio-Rad DC) detected the concentration of protein. Five milligrams per milliliter of protein and 1 μg/μL anti-CagA (sc-17450, Santa Cruz Biotechnology) was incubated overnight at 4°C, then added 50 μL 50% protein A/G-sepharose bead suspension for 2 h, and washed three times with pre-cold RIPA buffer, and added 50 μL protein sample buffer to collect in each tube, and proteins detected by western blotting analysis.

### Puncta formation assays

AGS cells were transfected with *GFP-MAP1LC3B* or *RFP-MAP1LC3B* plasmid for 24 h, following *H. pylori* infection for another 24 h. Radiance 2000 laser scanning confocal microscope detected the images of the cells, and image analysis with LaserSharp 2000 software (Bio-Rad, San Francisco, CA) as our previous study (Tang et al., 2016). According to methods for monitoring GFP-LC3 and mRFP-GFP-LC3 puncta formation assays (Klionsky et al., 2007; Mizushima et al., 2010), the average number of MAP1LC3B puncta per cell in *GFP-MAP1LC3B* or *RFP-MAP1LC3B*-positive cells (200 cells per sample) was determined (Pattingre et al., 2005).

### Cell viability

AGS cell viability was assessed using an MTT assay (Sigma) according to the manufacturer's instructions. Five milligrams per liter of MTT was added to each wells of AGS cells for 1–2 h, and dissolved in MTT solubilization solution. The absorbance at 590 nm (A590) was determined for each well using a microplate reader (Bio-Rad). After subtracting the background absorbance, the A590 value of the treated cells was divided by that of the untreated cells to determine the percentage of viable cells.

### MDC and AO staining assays

Monodansylcadaverine (MDC) and acridine orange (AO) staining was used to quantify the number of autolysosomes in AGS cells. Following treatment with *H. pylori* or transfection with plasmids/siRNAs, cells were stained with 10 mM MDC (sigma, 30432) and 1 mg/mL AO solution (sigma, A8097) at 37°C for 10 min, and fixed in 3% paraformaldehyde in PBS for 30 min. Photographs were obtained with a Radiance 2000 laser scanning confocal microscope (MDC, excitation wave length about 380 nm and emission filter 525 nm; AO, emission peak at 650 nm). The cells were then trypsinised and quantified by flow cytometry using a FACScan cytometer and CellQuest software (BD, New Jersey, USA). The percentage of cells with characteristic MDC or AO staining over the total cells was assessed.

### Transmission electron microscopy

AGS cells or gastric biopsy sections were collected and fixed in 2% paraformaldehyde, 0.1% glutaraldehyde and 0.1 M sodium cacodylate buffer (pH 7.4) for 2 h, then post-fixed in 1% OsO4, 0.5% potassium ferricyanide in cacodylate buffer for 1.5 h, then dehydrated with graded alcohol, and embedded in straight resin. Ultrathin sections were counterstained with 0.3% lead citrate and detected by Philips EM420 electron microscope. The method of counting autophagosomes' numbers was followed as described previously by Yla-Anttila et al. (2009). Data obtained by scoring for the presence of autophagic vacuoles (autophagosomes, autolysosomes) profiles per cell profile on the sections, and a total of 35 cells were recorded for triplicate samples per condition per experiment.

### Statistical analyses

The Student *t*-test was used to analyze between two groups, and one-way analysis of variance (ANOVA) was used to analyze among multiple group data, and expressed as mean ± standard error (SEM). GraphPad Prism software (GraphPad, San Diego, CA) was used for all statistical analyses. For all inferential statistics a *P* < 0.05 was considered significant.

## Results

### Autophagy is down-regulated in human gastric mucosa with CagA positive *H. pylori*

The clinical characteristics of 117 patients with (106) and those without (11) *H. pylori* infection are shown in Supplementary Table 2. *H. pylori* was successfully isolated from 106 patients, and genotyping for *cagA* and *vacA*. All *H. pylori* strains carry the *vacA* gene. To exclude the effect of VacA, the toxigenic *vacA* genotype (*vacA*^*s1m1*^), expressing a functional VacA toxic, were excluded from the study. In order to ensure that approximately equal numbers of each group, three equal groups were created for analyzing via random sampling methods, including normal control (8 cases), *cagA*^−^/*vacA*^*s1m2*^ (7 cases), *cagA*^+^/*vacA*^*s1m2*^ (8 cases).

**Figure 1:**
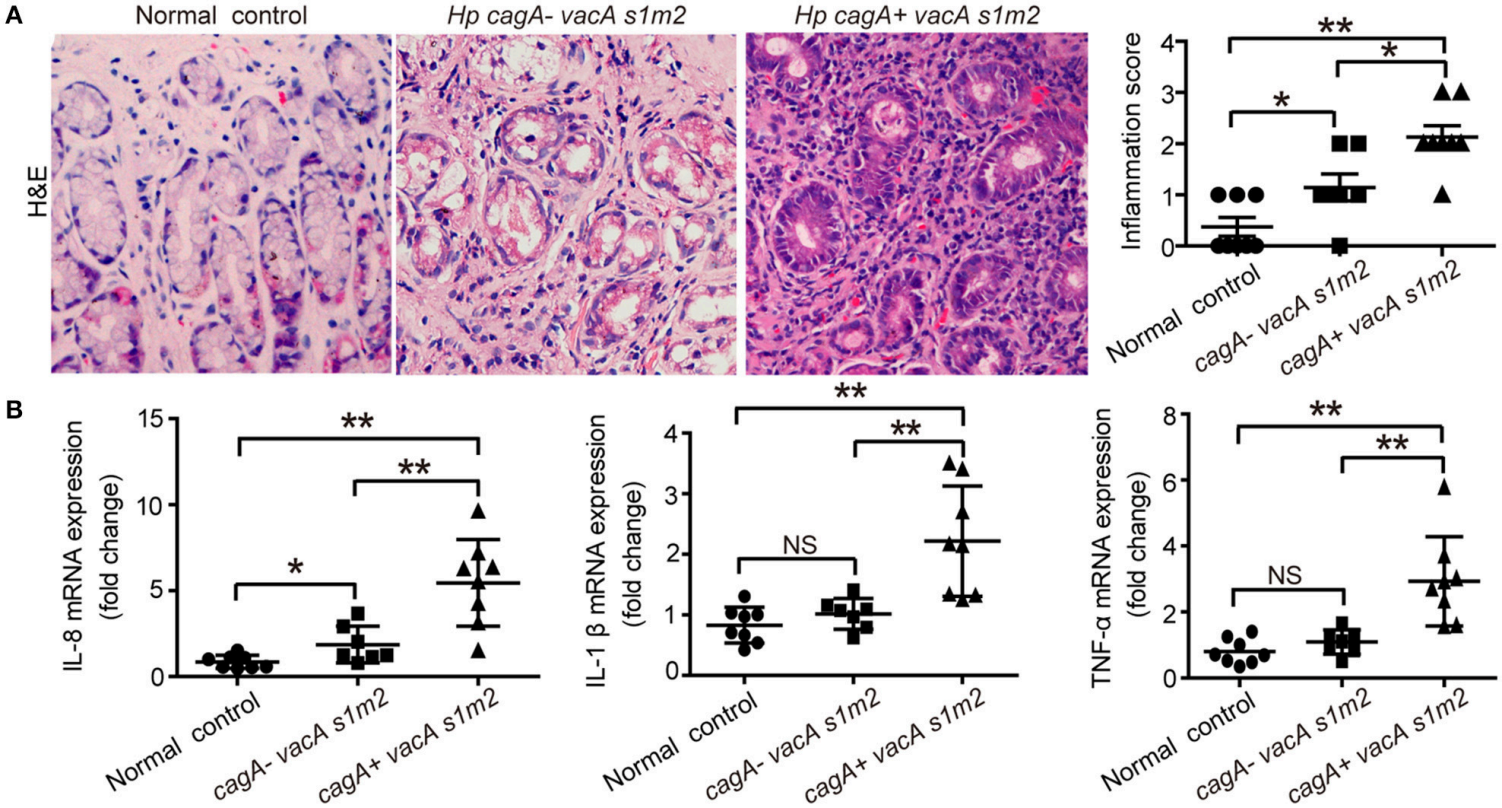
The inflammatory response on gastric biopsies from patients infected with *cagA*^−^/*vacA*^*s1m2*^ or *cagA*^+^/*vacA*^*s1m2*^ strains of *H. pylori*. **(A)** Histological scores of inflammation (H&E staining) in the gastric mucosa of patients without *H. pylori* infection and those infected with *cagA*^−^/*vacA*^*s1m2*^ or *cagA*^+^/*vacA*^*s1m2*^ strains of *H. pylori*. The intensity of staining is shown in the right graph and the data are expressed as mean±SEM. **(B)** mRNA expression of proinflammatory cytokines in gastric mucosa of patients without *H. pylori* (*n* = 8), patients infected with *cagA*^−^/*vacA*^*s1m2*^ (*n* = 7), and those infected with *cagA*^+^/*vacA*^*s1m2*^ (*n* = 8). All real-time PCR data are normalized to β-actin and expressed as fold change. Experiments performed in triplicate showed consistent results. ^*^P < 0.05, or ^**^P < 0.01.

To verify the effect of CagA in severe tissue inflammation, we evaluated the level of inflammation in gastric mucosa. Firstly, the degree of gastric inflammation was higher in patients infected with *cagA*^+^/*vacA*^*s1m2*^ strains than in those infected with *cagA*^−^/*vacA*^*s1m2*^ strains (Figure 1A). Notably, the mRNA levels of IL-8, TNF-α, and IL-1β in the gastric epithelial cells were significantly higher in patients infected with *cagA*^+^/*vacA*^*s1m2*^ strains than in patients without *H. pylori* infection or those infected with *cag*^−^/*vacA*^*s1m2*^ strains (Figure 1B).

**Figure 2:**
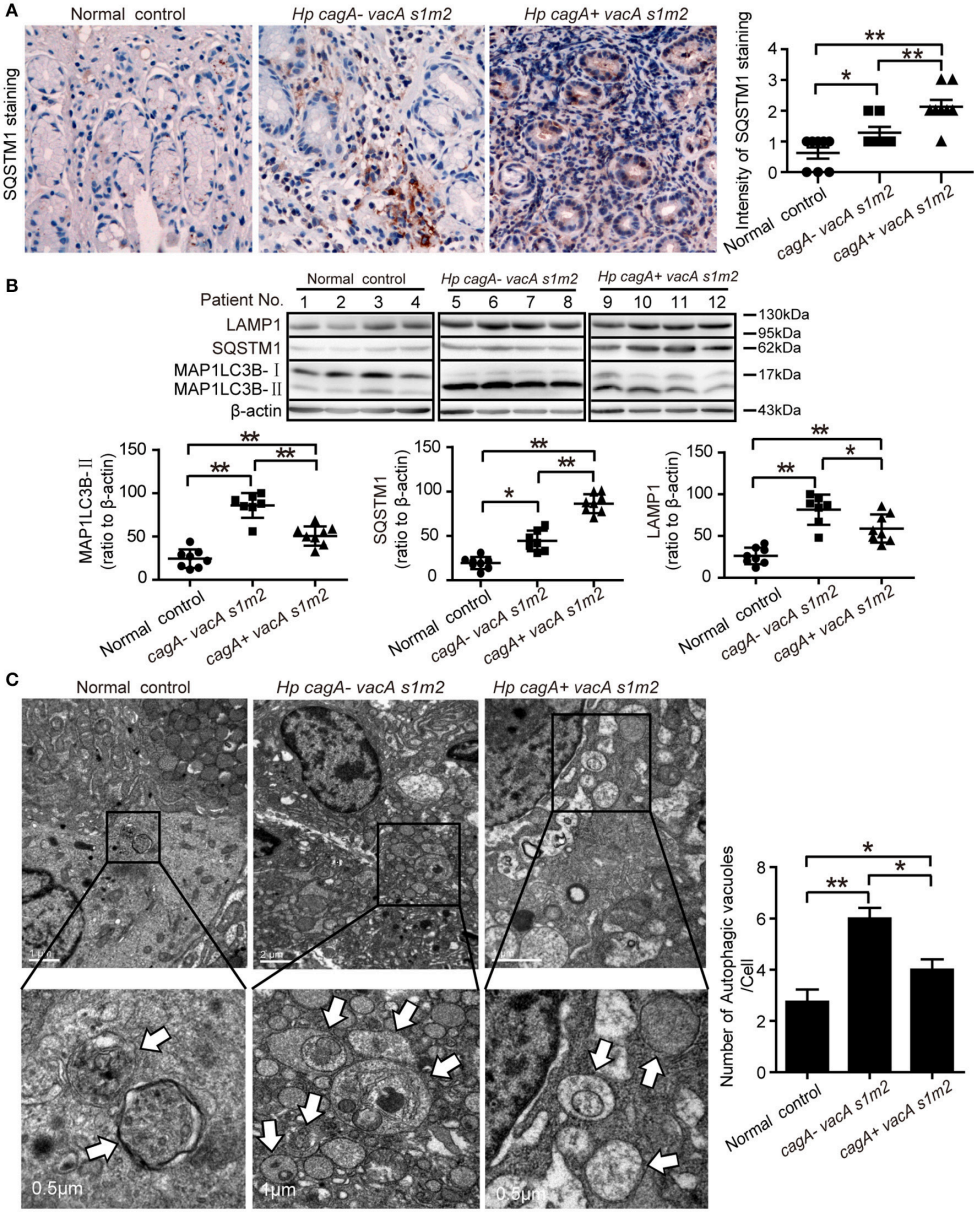
Autophagy is down-regulated in human gastric mucosa of patients infected with CagA positive *H. pylori* strains. **(A)** Immunohistochemistry showing SQSTM1 expression in the gastric mucosa of patients without *H. pylori* infection and those infected with *cagA*^−^/*vacA*^*s1m2*^ or *cagA*^+^/*vacA*^*s1m2*^ strains of *H. pylori*. The intensity of staining is shown in the right graph and the data are expressed as mean ± SEM. **(B)** Western blot assay showing the protein levels of MAP1LC3B-II, SQSTM1 and LAMP1 in the gastric mucosa of patients of normal control (patients 1–4), *cagA*^−^/*vacA*^*s1m2*^(patients 5–8), and *cagA*^+^/*vacA*^*s1m2*^ (patients 9–12) with the rates to β-actin being illustrated in the graphs in which the data are expressed as mean ± SEM. **(C)** Transmission electron microscopy showing autophagosomes in gastric biopsy sections of patients without *H. pylori* infection and those infected with *cagA*^−^/*vacA*^*s1m2*^ or *cagA*^+^/*vacA*^*s1m2*^ strains of *H. pylori*. Normal controls are patients without *H. pylori* infection. The white arrows indicate the autophagosomes. The numbers of autophagic vacuoles per cell in each TEM section (*n* = 35 cells) are shown in the right graph and the data are expressed as mean ± SEM. Experiments performed in triplicate showed consistent results. ^*^P < 0.05, or ^**^P < 0.01.

Furthermore, we evaluate the autophagic activity in gastric mucosal tissues from patients infected with different genotypes *H. pylori*. The SQSTM1/p62 (sequestosome1) protein serves as a link between LC3 and ubiquitinated substrates (Wang et al., 2010). Dysfunctional autophagy could result in an accumulation of SQSTM1, which has been involved in promoting inflammation (Raju et al., 2012). Therefore, we detected the levels of SQSTM1 in human gastric biopsies. As shown in Figure 2A, accumulation of SQSTM1 in the gastric biospy with *H. pylori* infection was significantly higher than those uninfected normal control, and the accumulation of SQSTM1 was significantly higher in the gastric epithelium cells in patients infected with *cagA*^+^/*vacA*^*s1m2*^ strains than in those infected with *cagA*^−^/*vacA*^*s1m2*^ strains (*P* < 0.001) as determined by immunohistochemistry. Moreover, in patients infected with *cagA*^−^/*vacA*^*s1m2*^ strains, the ratio of microtubule-associated protein 1 light chain 3 beta-II (MAP1LC3B-II) to β-actin and the lysosomal-associated membrane protein 1 (LAMP1, the late endosomal lysosomal marker) protein levels was higher than that in patients infected with *cagA*^+^/*vacA*^*s1m2*^ strains, and the SQSTM1 protein levels increased in patients infected with CagA-positive *H. pylori* (Figure 2B). In addition, infection of *cagA*^+^/*vacA*^*s1m2*^ strains was significantly associated with increased levels of mRNA expression of SQSTM1 (Supplementary Figure 1A), but not of BECN1 (Supplementary Figure 1B). Furthermore, it was also revealed an increase in the number of autophagosomes in patients infected with *cagA*^−^/*vacA*^*s1m2*^ strains compared with that in *cagA*^+^/*vacA*^*s1m2*^ group in TEM analysis (Figure 2C). Both *cagA*^+^/*vacA*^*s1m2*^ and *cagA*^−^/*vacA*^*s1m2*^ groups displayed high autophagy activity than the normal control group. These findings indicate that *H. pylori* infection could induce inflammation response and autophagy activity in the gastric epithelium cells in *vivo*, but CagA-positive *H. pylori* are associated with more severe inflammation, and down-regulates autophagic response *in vivo*.

### CagA could inhibit the generation of autophagosomes in AGS cells

**Figure 3:**
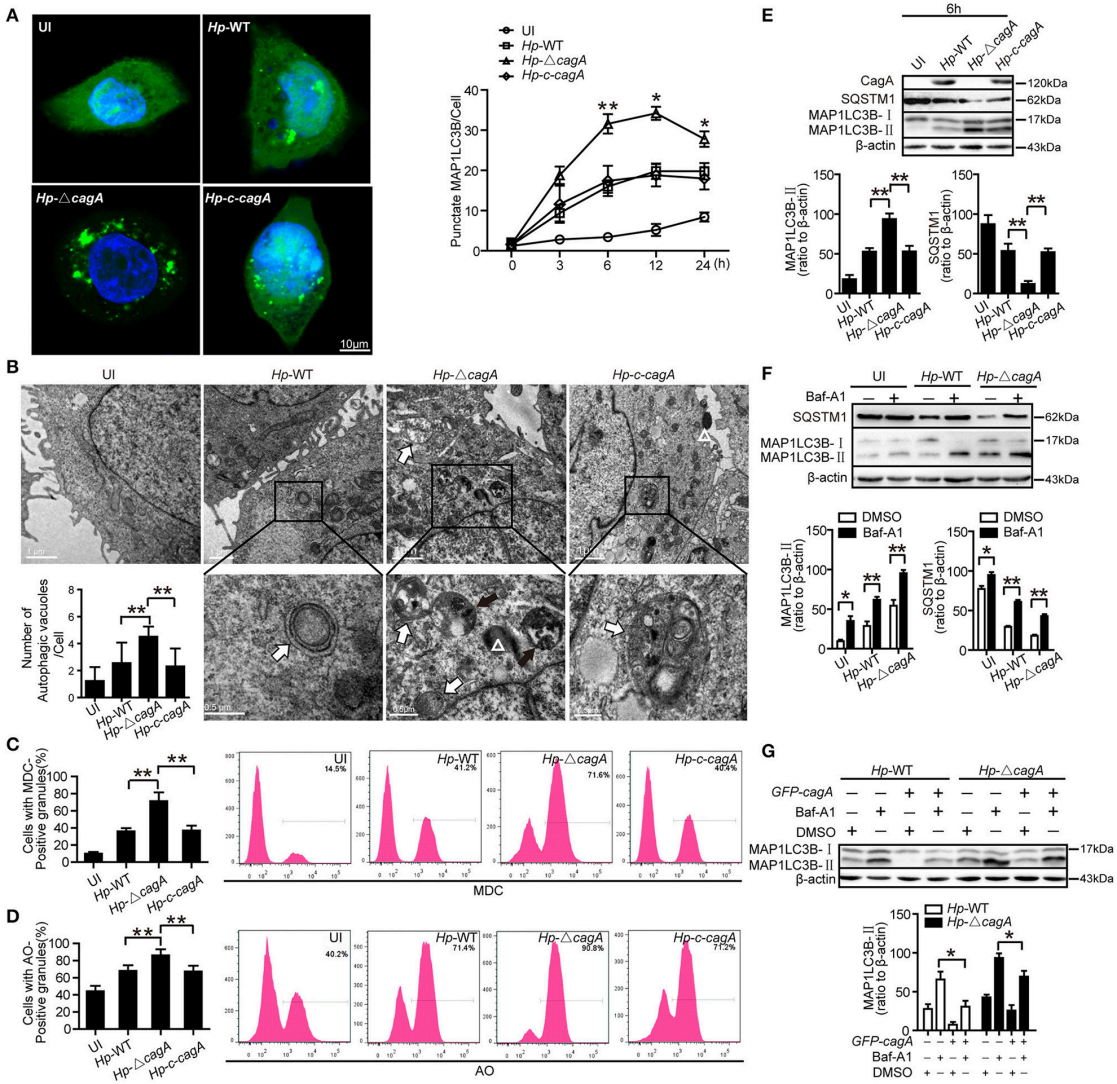
CagA could inhibit the generation of autophagosomes in AGS cells. **(A)** Confocal microscopy showing AGS cells transfected with *GFP-MAP1LC3B* without *H. pylori* infection (UI), and transfected AGS cells with the wild type *H. pylori* (*Hp*-WT), the *cagA*-knockout *H. pylori* (*Hp*-Δ*cagA*) or the *cagA*-knockout complementation mutant *H. pylori* (*Hp*-*c-cagA*) (MOI = 100:1) infection for 6 h (left) and the indicated periods of time (right). Scale bars: 10 μm. The number of *GFP-MAP1LC3B* puncta in each cell (*n* ≥ 200 cells) was counted. **(B)** Representative transmission electron microscopy showing AGS cells without *H. pylori* infection and those infected with *Hp*-WT, *Hp*-Δ*cagA*, or *Hp*-*c-cagA* (MOI = 100:1) for 6 h. The white arrows indicate autophagosomes, and the black arrows indicate autolysosomes, and the white triangle indicate *H. pylori*. The numbers of autophagic vacuoles per cell in each TEM section (*n* = 35 cells) are shown in the lower left graph and the data are expressed as mean ± SEM. **(C,D)** Flow cytometry showing MDC and AO staining of AGS cells 6 h after infection with *Hp*-WT, *Hp*-Δ*cagA*, or *Hp*-*c-cagA* (MOI = 100:1). **(E)** Western blotting showing the protein levels of CagA, SQSTM1, and MAP1LC3B-II with the rates of SQSTM1 and MAP1LC3B-II to β-actin in AGS cells infected with *Hp*-WT, *Hp*-Δ*cagA*, or *Hp*-*c-cagA* (MOI = 100:1) for 6 h. **(F)** Measurement of MAP1LC3B-II conversion and SQSTM1 in AGS cells infected with *Hp*-WT or *Hp*-Δ*cagA* (MOI = 100:1) for 6 h in the presence of Baf-A1 (10 nM). **(G)** AGS cells were transfected with *GFP-CagA*, and then infected with *Hp*-WT or *Hp*-Δ*cagA* (MOI = 100:1) for 6 h in the presence of Baf-A1 (10 nM). Results shown are representative of three independent experiments. ^*^P < 0.05, ^**^P < 0.01.

To further validate the role of CagA in autophagy regulation, we next infected AGS cells with the *H. pylori* wide-type (*Hp*-WT), *H. pylori cagA*-knockout mutant (*Hp*-Δ*cagA*) or *H. pylori cagA*-knockout complementation (*Hp*-*c*-*cagA*) (MOI = 100:1), which strains the expression of VacA is similar during infection (Supplementary Figure 2A), and evaluated the kinetics of autophagosome formation by a *GFP-MAP1LC3B* puncta formation assay. Formation of MAP1LC3B puncta, peaked at 12 h and decreased at 24 h (Figure 3A and Supplementary Figure 2B). And compared with cells infected with *Hp-*WT or *Hp*-*c*-*cagA*, there was a significantly increased percentage of cells with formation of MAP1LC3B puncta for cells infected with *Hp*-Δ*cagA* (Figure 3A). TEM revealed an increase in the number of autophagic vacuoles (autophagosomes and autolysosomes) in AGS cells infected with *Hp*-Δ*cagA*-infected cells, compared with cells infected with *Hp-*WT or *Hp*-*c*-*cagA* (Figure 3B). Similar results were obtained in MDC (Figure 3C and Supplementary Figure 2D) and AO (Figure 3D and Supplementary Figure 2D) staining. Additionally, *Hp*-Δ*cagA* induced MAP1LC3B-II formation, and decreased SQSTM1 protein expression at a higher level, compared with *Hp-*WT or *Hp*-*c*-*cagA*, at 6, 12, and 24 h (Figure 3E and Supplementary Figure 2C). Furthermore, inhibition of autophagy by Baf-A1 challenge resulted in further accumulation of both MAP1LC3B-II and SQSTM1 in AGS cells after 6 h of *Hp-*WT or *Hp*-Δ*cagA* infection (Figure 3F), suggesting that *H. pylori* CagA did not inhibit the fusion of autophagosomes with lysosomes. Furthermore, under *Hp*-WT or *Hp-*Δ*cagA* infection, the levels of MAP1LC3B-II in AGS cells transfected with the CagA expression plasmid (*GFP-CagA)* were decreased in comparison to that in transfected-control cells (Figure 3G), suggesting that over-expression of CagA lead to further reduction of autophagic flux. Collectively, these data suggest that *H. pylori* CagA may inhibit the generation of autophagosomes in AGS cells.

### CagA down-regulates starvation-induced autophagy in AGS cells

**Figure 4:**
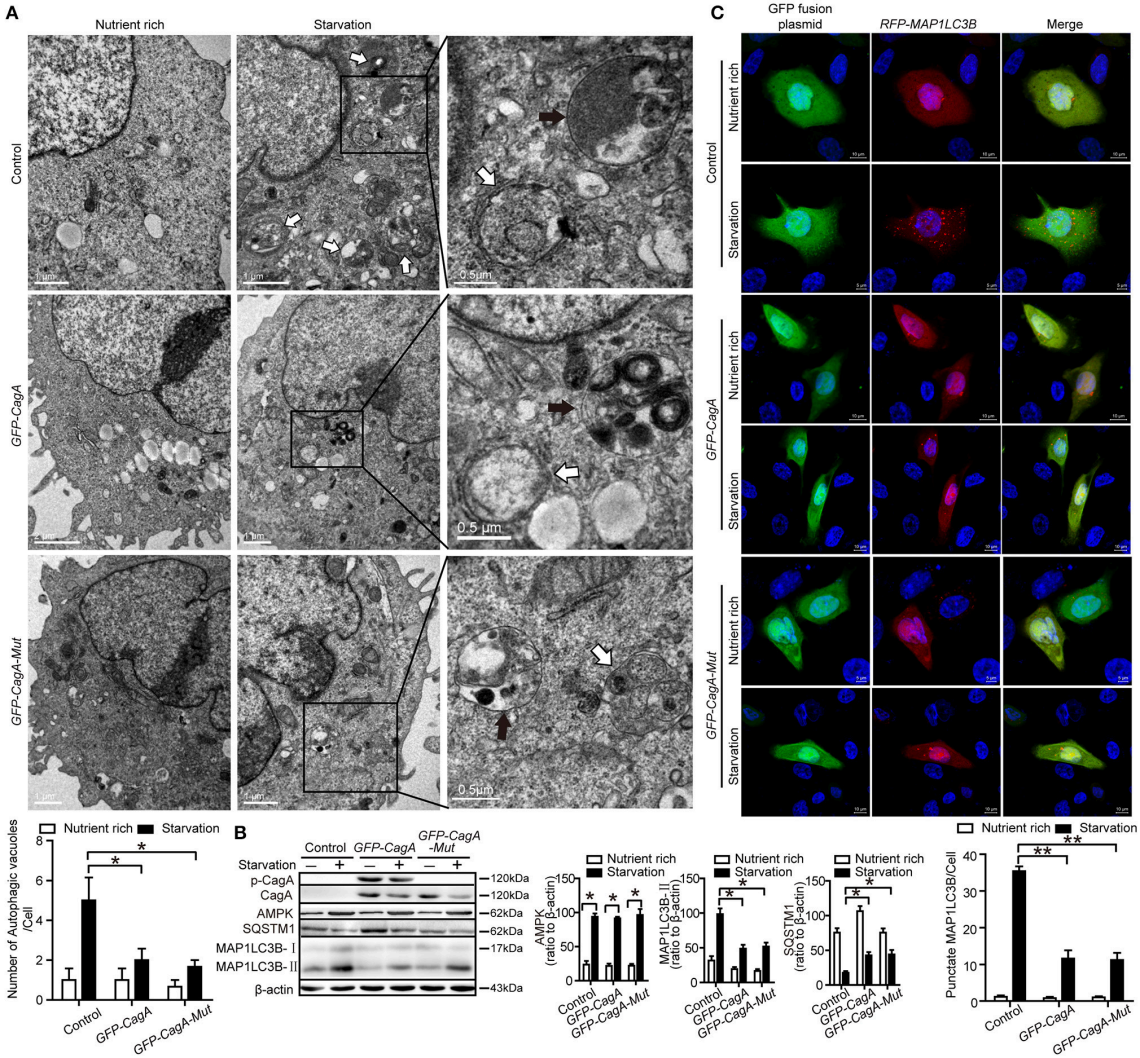
CagA down-regulates starvation-induced autophagy in AGS cells. **(A)** Transmission electron microscopy showing autophagic vacuoles in AGS cells transfected with *GFP-CagA, GFP-CagA-Mut*, or a control (*pEGFP-C1*) plasmid before pretreatment of normal media or subjected to 4 h starvation. The white arrows indicate autophagosomes, and the black arrows indicate autolysosomes. The numbers of autophagic vacuoles per cell in each TEM section (*n* = 35 cells) are shown in the lower graph and the data are expressed as mean ± SEM. **(B)** Western blot assay showing MAP1LC3B-II conversion and expression of p-CagA, CagA, AMPK, and SQSTM1 in AGS cells transfected with *GFP-CagA, GFP-CagA-Mut*, or a control (*pEGFP-C1*) plasmid in the nutrient rich medium or 4 h starvation. **(C)** Confocal microscopy showing AGS cells co-transfected with *RFP-MAP1LC3B* and *GFP-CagA, GFP-CagA-Mut*, or a control (*pEGFP-C1*) plasmid in the nutrient rich medium or 4 h starvation. Scale bars: 5 or 10 μm. The number of *RFP-MAP1LC3B* puncta in each cell (*n* ≥ 200 cells) was counted. Experiments were performed in triplicate, and all replicates showed similar results. ^*^P < 0.05, ^**^P < 0.01.

In order to eliminate the influence of *H. pylori* itself on autophagy, starvation-triggered autophagy was performed in AGS cells after transfecting the CagA expression plasmid (*GFP-CagA)* or tyrosine phosphorylation point mutant of CagA plasmid (*GFP-CagA-Mut*). At least 50% transfection efficiency was achieved for transfection of *GFP-CagA* and *GFP-CagA-Mut* in AGS (Supplementary Figure 3A). Although cell viability was influenced by starvation to a certain extent during the first 4 h, it appears not to be significantly influenced afterwards (Supplementary Figure 3B). During nutrient starvation, in the AGS cells transfected with *GFP-CagA* or *GFP-CagA-Mut*, there was a significant decrease in the number of autophagosomes as determined by TEM, compared with cells transfected with control plasmid (*P* < 0.05, Figure 4A). The ratio of MAP1LC3B-II to β-actin was also significantly decreased in cells transfected with *GFP-CagA* or *GFP-CagA-Mut* following starvation treatment (*P* < 0.05, Figure 4B). Similarly, SQSTM1 expression was increased in cells transfected with *GFP-CagA* or *GFP-CagA-Mut* following starvation treatment (*P* < 0.05, Figure 4B). Interestingly, we observed that transfection with *GFP-CagA* or *GFP-CagA-Mut* had no effect on the expression of the AMP activated protein kinase (AMPK, an energy sensor; Figure 4B), indicating that CagA suppressed starvation-induced autophagy may not via the AMPK signal pathway. Furthermore, as shown in Figure 4C, the number of *RFP-MAP1LC3B* puncta in AGS cells co-transfected with *RFP-MAP1LC3B* and *GFP-CagA* was decreased after starvation treatment (*P* < 0.05). Taken together, these results suggest that CagA suppressed starvation-induced autophagy, which may not be dependent on tyrosine phosphorylation of CagA.

### Autophagy inhibition increases cytokines production

**Figure 5:**
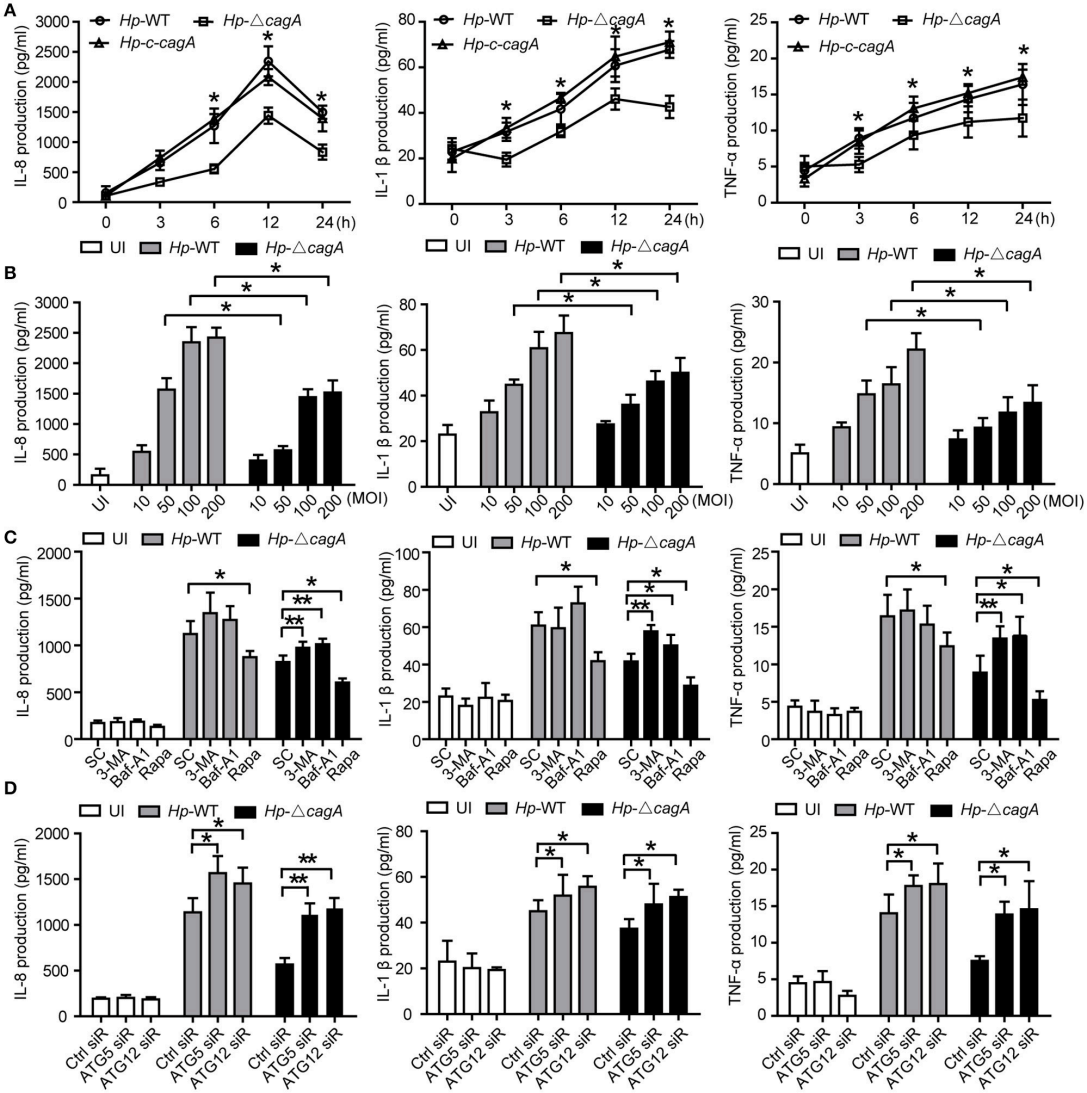
Inhibition of autophagy enhances cytokines production induced by the cagA-knockout *H. pylori*. **(A,B)** Production of IL-8, IL-1β and TNF-α in AGS cells infected *Hp*-WT, *Hp*-Δ*cagA* or *Hp*-*c*-*cagA* at MOI of 100 for the indicated periods of time **(A)** or at different MOIs (10, 50, 100, and 200) for 12 h **(B)**, as assessed by enzyme-linked immunosorbent assay (ELISA). **(C)** After pretreatment of SC (solvent control, 0.1% DMSO), 3-MA (2 mM), Baf-A1 (10 nM) or Rapa (100 nM), AGS cells were infected with *Hp*-WT or *Hp*-Δ*cagA* (MOI = 100:1) for 6 h. Supernatants were assessed by ELISA for levels of IL-8, IL-1β, and TNF-α. **(D)** Production of IL-8, IL-1β, and TNF-α in AGS cells transfected with siRNA specific for ATG5 or ATG12 (50 nM) for 24 h and infected with *Hp*-WT or *Hp*-Δ*cagA* (MOI = 100) for 6 h, as assessed by ELISA. Data are presented as the mean ± SEM of three experiments. ^*^P < 0.05, ^**^P < 0.01.

To clarify the role of CagA in the inflammation, the expression of proinflammatory cytokines (IL-8, TNF-α, and IL-1β), which are involved in gastritis during *H. pylori* infection (Nakachi et al., 2000), was examined by ELISA assay. These cytokines were significantly higher in AGS cells infected with *Hp*-WT or *Hp*-*c*-*cagA* than in those infected with *Hp*-Δ*cagA* at different time points (Figure 5A). Moreover, These cytokines in AGS cells infected with *Hp-*WT and *Hp*-Δ*cagA* was increased following the increase of the bacterial load. AGS cells infected with *Hp-*WT produced greater amounts of the cytokines than cells infected with *Hp*-Δ*cagA* (Figure 5B).

We also examined the production of the cytokines with the autophagy enhancer (Rapa, Rapamycin) or inhibitors (3-MA or Baf-A1) treatment during *Hp*-WT and *Hp*-Δ*cagA* infection. The effects of two autophagy inhibitors, and one enhancer, are shown in Supplementary Figures 4A–C. Autophagy inhibitors significantly increased the cytokines and activated NF-κB, and enhancer Rapa decreased the ones in AGS cells infected *Hp*-Δ*cagA* infection (Figure 5C and Supplementary Figure 4E). After 24 h infection, the three proinflammatory cytokines were increased with the inhibitors in cells infected with *Hp*-WT and *Hp*-Δ*cagA* (Supplementary Figure 4F). Moreover, the production of proinflammatory cytokines and activity of NF-κB were significantly increased in AGS cells transfected with siRNAs for ATG5 or ATG12 upon *H. pylori* infection (Figure 5D and Supplementary Figure 4G). These data suggested that autophagy plays a critical role in the inflammation induced by *H. pylori*.

### c-Met is an important adaptor in CagA-mediated autophagy pathway

**Figure 6:**
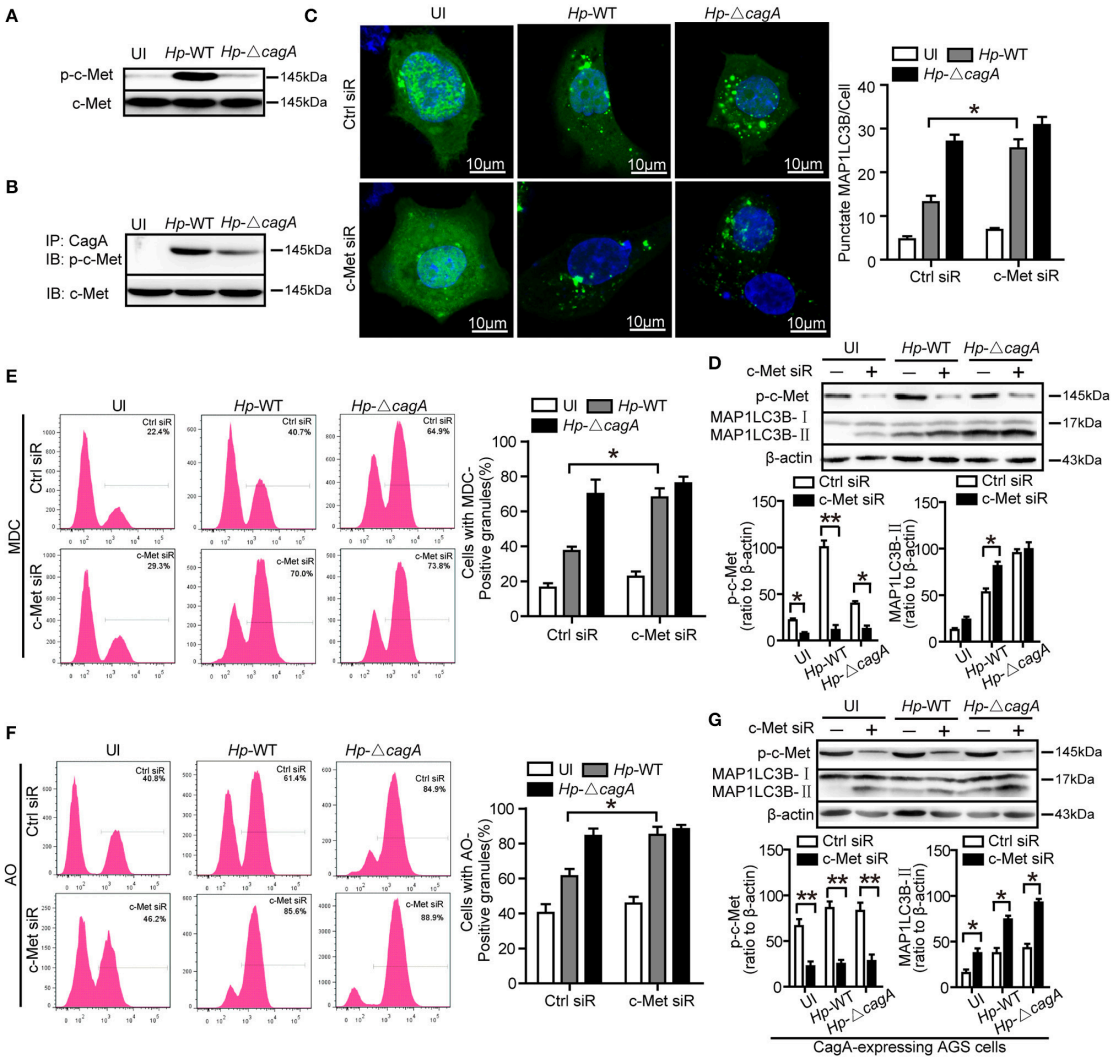
c-Met is an important adaptor in CagA-mediated autophagy pathway. **(A,B)** AGS cells were infected with *Hp-*WT or *Hp-*Δ*cagA*, and p-c-Met and c-Met were detected by western blot. CagA was immunoprecipitated from lysates. Immunoprecipitates (IP) were subjected to SDS-PAGE and immunoblot (IB) analysis with anti-p-c-Met (top) or anti–c-Met (bottom) antibodies. **(C)** Confocal microscopy showing AGS cells co-transfected with *GFP-MAP1LC3B* plasmid and c-Met siRNAs or control siRNA for 24 h, and then infected with *Hp*-WT or *Hp*-Δ*cagA* for 6 h. The percentages of cells with MAP1LC3B punctas are shown in the right graph with data being expressed as means ± SEM of three experiments (*n* ≥ 200 cells). **(D)** Western blot analysis of p-c-Met, MAP1LC3B-II conversion and β-actin in AGS cells transfected with c-Met siRNA or control siRNA and infected with *Hp*-WT or *Hp*-Δ*cagA* for 6 h. p-c-Met and MAP1LC3B-II band intensity was normalized to β-actin. **(E,F)** Flow cytometry showing MDC (upper panel) and AO (lower panel) staining of AGS cells transfected with c-Met siRNA or control siRNA and then infected with *Hp*-WT or *Hp*-Δ*cagA* for 6 h. **(G)** Western blot analysis of p-c-Met, MAP1LC3B-II conversion and β-actin in CagA-expressing AGS cells (AGS cells after transfecting the CagA expression plasmid, *GFP-CagA*) after transfected with c-Met siRNA or control siRNA and infected with *H. pylori* as described above. Experiments performed in triplicate showed consistent results. ^*^P < 0.05, ^**^P < 0.01.

The previous study reported that CagA has been known to activate c-Met and the PI3K/AKT pathway (Churin et al., 2003). However, it is not clear whether c-Met could regulate autophagy. The wild type *H. pylori* infection activated c-Met in AGS cells (Figure 6A). CagA was coimmunoprecipitated with c-Met in AGS cells infection with *Hp-*WT (Figure 6B). This result was consistent with previous study (Oliveira et al., 2009). The effects of c-Met depletion through siRNA interference are shown in Supplementary Figure 4D. The number of *GFP-MAP1LC3B* puncta in c-Met siRNA group was higher than that of control group upon infection with the *Hp*-WT (*P* < 0.05, Figure 6C). It was a significant increase in the ratio of MAP1LC3B-II to β-actin in c-Met siRNA group than in the control siRNA upon infection with the *Hp*-WT (*P* < 0.05, Figure 6D). Furthermore, MDC and AO staining showed that c-Met siRNA group induced the formation of autophagolysosomes in AGS cells at a significantly higher level, compared with control siRNA group in AGS cells infected with *Hp*-WT (*P* = 0.008 and 0.018, respectively, Figures 6E,F and Supplementary Figure 5A). Moreover, in CagA-expressing AGS cells, the ratio of MAP1LC3B-II to β-actin significantly increased by c-Met siRNA regardless of infection status (Figure 6G). These results demonstrate that c-Met may be an important adaptor in CagA-mediated autophagy pathway.

### CagA regulates autophagy through c-Met/Akt signaling pathway

**Figure 7:**
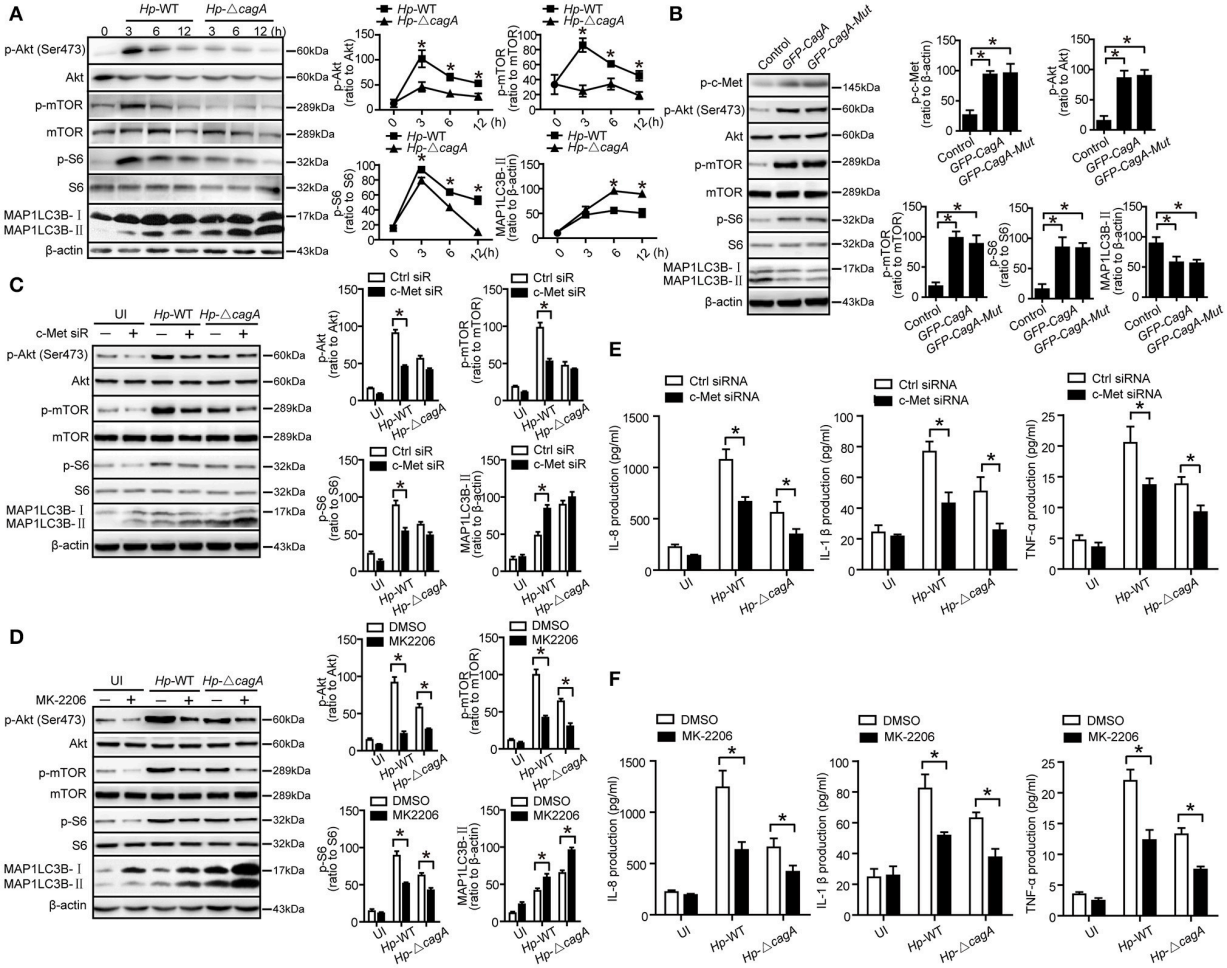
CagA regulates autophagy through PI3K/AKT/mTOR pathway. **(A)** Expression of p-Akt(Ser473), Akt, p-mTOR, mTOR, p-S6, S6, MAP1LC3B-I, and MAP1LC3B-II in AGS cells infected with *Hp*-WT or *Hp*-Δ*cagA*(MOI = 100) at different time points. **(B)** Expression of p-Akt(Ser473), Akt, p-mTOR, mTOR, p-S6, S6, MAP1LC3B-I, and MAP1LC3B-II in AGS cells transfected with *GFP-CagA, GFP-CagA-Mut* or control plasmid. **(C)** Expression of p-Akt(Ser473), Akt, p-mTOR, mTOR, p-S6, S6, MAP1LC3B-I, and MAP1LC3B-II in AGS cells transfected c-Met siRNA or control siRNA (50 nM) for 24 h and then infected with *Hp*-WT or *Hp*-Δ*cagA* (MOI = 100) for 6 h. **(D)** Expression of p-Akt(Ser473), Akt, p-mTOR, mTOR, p-S6, S6, MAP1LC3B-I, and MAP1LC3B-II in AGS cells infected with *Hp*-WT or *Hp*-Δ*cagA* (MOI = 100) for 6 h with or without pre-treatment of MK-2206 (8 μM). **(E)** Production of IL-8, IL-1β and TNF-α in AGS cells transfected c-Met siRNA or control siRNA (50 nM) for 24 h and then infected with *Hp*-WT or *Hp*-Δ*cagA* (MOI = 100) for 6 h, as assessed by ELISA. **(F)** After pretreatment of DMSO or MK-2206 (8 μM), AGS cells were infected with *Hp*-WT or *Hp*-Δ*cagA* (MOI = 100:1) for 6 h. Supernatants were assessed by ELISA for levels of IL-8, IL-1β, and TNF-α. Experiments performed in triplicate showed consistent results. ^*^P < 0.05.

Given that c-Met could activate PI3K/AKT/mTOR pathway (Lim and Walikonis, 2008; Tang et al., 2015), we hypothesized that PI3K/AKT/mTOR pathway might play an important role in the process of CagA-mediated autophagy. We analyzed the activation status of the key members of autophagy-related PI3K/Akt/mTOR pathways. As shown in Figure 7A, *Hp-*WT activated Akt kinase at Ser-473 site at a significantly higher level than did *Hp*-Δ*cagA* (*P* = 0.018), which was consistent with a previous report (Tabassam et al., 2009). Both *Hp*-WT and *Hp*-Δ*cagA* increased MAP1LC3B-II expression, but *Hp*-Δ*cagA* did at a significantly higher level than did *Hp*-WT (Figure 7A). There was a significant increase in the levels of phosphorylated mTOR (p-mTOR) and phosphorylated S6 ribosomal protein (p-S6) upon *Hp-*WT vs. *Hp*-Δ*cagA* (Figure 7A). Tyrosine phosphorylation of CagA did not affect the expression levels of proteins related to PI3K/Akt/mTOR pathway and autophagy in AGS cells (Figure 7B), whereas c-Met siRNA significantly decreased the level of p-Akt, p-mTOR, and p-S6, and increased MAP1LC3B-II levels (Figure 7C). Moreover, treatment of MK-2206, a specific inhibitor of Akt, abrogated Akt activation, and reversed the ratio of MAP1LC3B-II/β-action, and decreased the level of p-Akt, p-mTOR, and p-S6 (Figure 7D). Then, to investigate whether c-Met siRNA or MK-2206 reverse inflammatory response during *H. pylori* infection, we detected the expression of inflammatory cytokines. As shown in Figure 7E, there was a significant decrease in the production of proinflammatory cytokines in cells transfected with siRNA specific for c-Met upon infection. Similarly, the expression of inflammatory cytokines significantly decreased in AGS cells treated with MK2206 during infection (Figure 7F). Together, we concluded that the CagA-mediated autophagy pathway may be dependent on the c-Met/Akt signaling pathway, which could regulate the expression of inflammatory cytokines.

## Discussion

In the present study, we observed that (i) autophagy was down-regulated in gastric mucosal tissues infected with *cagA*+ positive *H. pylori* strains, with increased gastric inflammation; (ii) CagA inhibited autophagy and induced production of proinflammatory cytokines in AGS cells; (iii) CagA downregulated starvation-induced autophagy; (iv) Inhibition of autophagy enhanced *H. pylori-*induced cytokine production; (v) c-Met siRNA significantly affected CagA-mediated autophagy; and (vi) CagA regulates autophagy through c-Met/Akt signaling pathway. These findings indicate that CagA may act as a negative regulator of autophagy in *H. pylori*-induced inflammatory response. Specifically, given that inflammation and autophagy are major determinants of gastric malignancy (Mohri et al., 2012), it also opens a new avenue of research on gastric malignancies, especially prophylaxis and treatment.

Autophagy, as the quality control of the cellular environment, plays an important role in the protective response during infection (Deretic, 2010). However, a number of pathogens could subvert autophagy to promote inflammation generation, the occurrence and promotion of tumor, and genetic instability (Deretic and Levine, 2009). Previous studies have reported that autophagosome formation was induced by VacA of *H. pylori in vitro* (Terebiznik et al., 2009), but VacA could also disrupt autophagic flux to promote the infection (Raju et al., 2012). In the present study, we demonstrated that CagA could inhibit autophagy, increased the production of proinflammatory cytokines and facilitated gastric inflammation. In gastric mucosal tissues, autophagy was downregulated in patients infected with CagA positive *H. pylori* strains, which was accompanied with an increased production of cytokines. To rule out the effect of VacA on autophagy, the toxigenic vacA genotype (*vacA*^*s1m1*^), expressing a functional VacA toxic, were excluded from the study. We selectively recruited patients with *H. pylori* negative infection, ones infected with *H. pylori cagA*^−^/*vacA*^*s1m2*^ strains and ones infected with *cagA*^+^/*vacA*^*s1m2*^ strains in the present study. As shown in Figures 2A–C, the signaling molecules such as, lower MAP1LC3B-II conversion, SQSTM1 accumulation and decreased LAMP1 expression (late endosomal/lysosomal marker; Yu et al., 2010) in gastric mucosal tissues infected with *cagA*^+^ *H. pylori* strains compared with *cagA*^−^ *H. pylori* strains, which indicated that autophagic activity was decreased with increased gastric inflammation in patients infected with *cagA*^+^/*vacA*^*s1m2*^ strains. These results suggest that *H. pylori* CagA might induce inflammation by inhibiting autophagy. The intracellular CagA could be degraded by autophagy and short lived in AGS cells (Tsugawa et al., 2012). These finding suggest that induction of autophagy by *H. pylori* infection can degrade CagA by host cell defenses. Our observations indicate that persistent infection of bacterial exerts CagA to inhibit autophagy and induce inflammation.

Our observation that CagA is a negative regulatory factor for autophagy induced by *H. pylori* infection is consistent with findings of Deen's study (Deen et al., 2015), which showed that cagPAI of *H. pylori* has an inhibitory role in autophagy in macrophages. In addition, our results are also consistent with another study in which gastric biopsies from patients infected with *cagA*^+^/*vacA*^*s1m1*^ strains showed a significantly higher accumulation of SQSTM1 in the gastric epithelium compared with patients infected with a nonfunctional *cagA*^−^/*vacA*^*s*2*m*2^ strains (Raju et al., 2012). Given that tyrosine phosphorylation of CagA plays critical roles in the activation of many pathways (Moss et al., 2001; Boonyanugomol et al., 2011; Wandler and Guillemin, 2012), we constructed the corresponding tyrosine phosphorylation mutants of the parent CagA (*GFP-CagA-Mut*). Our results demonstrated that tyrosine phosphorylation of CagA did not affect the PI3K/Akt/mTOR pathway, autophagy, and inflammation, suggesting that inhibition of autophagy is not dependent on tyrosine phosphorylation of CagA. Thus, the more specific mechanism of autophagy inhibited by CagA needs to be further investigated in the future.

It is well-established that autophagy plays critical roles in innate and adaptive immunity (Deretic et al., 2013), and disrupted autophagy is involved in secreting the proinflammatory cytokines, such as: IL-1α, IL-8, and IL-18 (Martins et al., 2015). Several studies have reported that autophagy may be an important mechanism for controlling inflammation in patients with Crohn's disease (Hampe et al., 2007; Rioux et al., 2007). Here, we demonstrated that autophagy inhibition enhanced the production of proinflammatory cytokines in *H. pylori* infection. SQSTM1, which is a major cargo ubiquitin-binding receptor in cells, is degraded by autolysosomes, and deficiencies of autophagy leads to accumulation of SQSTM1 (Wang et al., 2006). Moreover, SQSTM1 has further beneficial effects in NF-κB dependent cytokine production (Dupont et al., 2009). In the present study, there was a significant accumulation of SQSTM1 in the gastric mucosa of patients infected with CagA-positive *H. pylori* strains. When autophagy was inhibited, the activity of NF-κB was enhanced in AGS cells infected with mutant *H. pylori* strains (i.e., *Hp*-Δ*cagA*). These results suggested that autophagy inhibited by CagA leads to accumulation of SQSTM1, resulting in NF-κB dependent cytokine production.

CagA activates c-Met through its CRPIA (i.e., conserved repeat responsible for phosphorylation-independent activity) motif, which is critical for activation of PI3K/Akt signaling pathway and the pleiotropic transcriptional responses in *H. pylori* infection, including activation of NF-κB and β-catenin (Suzuki et al., 2009). Our data showed that, CagA was coimmunoprecipitated with c-Met in AGS cells during *H. pylori* infection, and siRNA silencing mediated c-Met knockdown in AGS enhanced the autophagy significantly in cells infected with wide-type *cagA*^+^ *H. pylori* strain (i.e., *Hp-*WT). The PI3K/Akt signaling pathway participates in autophagy via mTOR, an autophagic regulators, resulting in autophagy suppression (Harashima et al., 2012). In the present study, we showed that CagA-positive *H. pylori* significantly increased the level of phosphorylated Akt at Ser473 and the levels of p-mTOR and p-S6 in AGS cells. The Akt inhibitor reversed the ratio of MAP1LC3B-II/β-actin in CagA-positive *H. pylori* infection, and blocked the level of phosphorylated Akt at Ser473. These findings clearly indicate that CagA inhibits autophagy via the c-Met-PI3K/Akt-mTOR signaling pathway.

Although CagA has already been reported to be a virulent factor in the inflammation induced by *H. pylori* infection, this is a new study demonstrating that CagA negatively regulates autophagy through c-Met-PI3K/Akt-mTOR signaling pathway, which is associated with increased expression of proinflammatory cytokines. Therefore, we postulate that inhibition of autophagy by CagA promotes gastric inflammation, which, in turn, initiates the multistep of gastric carcinogenesis (Correa, 1992). Moreover, given the pleiotropic actions of CagA, the interplay between CagA and autophagy regulation mechanism, which needs to be further investigated. A better understanding of the molecular mechanisms by which *H. pylori* infection modulates and interplays with autophagy will shed new insight into the development of more effective therapeutic strategies for *H. pylori* infection.

## Author contributions

Conceived and designed the experiments: NL and BT. Performed the experiments: YJ, PZ, YZ, and YF. Analyzed the data: QL, KW, WZ, GG, and TW. Wrote the paper: YjF, BQ, XM, and QZ.

### Conflict of interest statement

The authors declare that the research was conducted in the absence of any commercial or financial relationships that could be construed as a potential conflict of interest.
