# Supplementary material for: Helicobacter pylori CagA Protein Negatively Regulates Autophagy and Promotes Inflammatory Response via c-Met-PI3K/Akt-mTOR Signaling Pathway
Source: Front Cell Infect Microbiol. 2017 Sep 21;7:417. doi: 10.3389/fcimb.2017.00417 (PMC5613121; doi:10.3389/fcimb.2017.00417)
Supplement: Supplementary file 1 [file DataSheet1.DOC]

**Supplementary Materials**

**Supplementary Data**


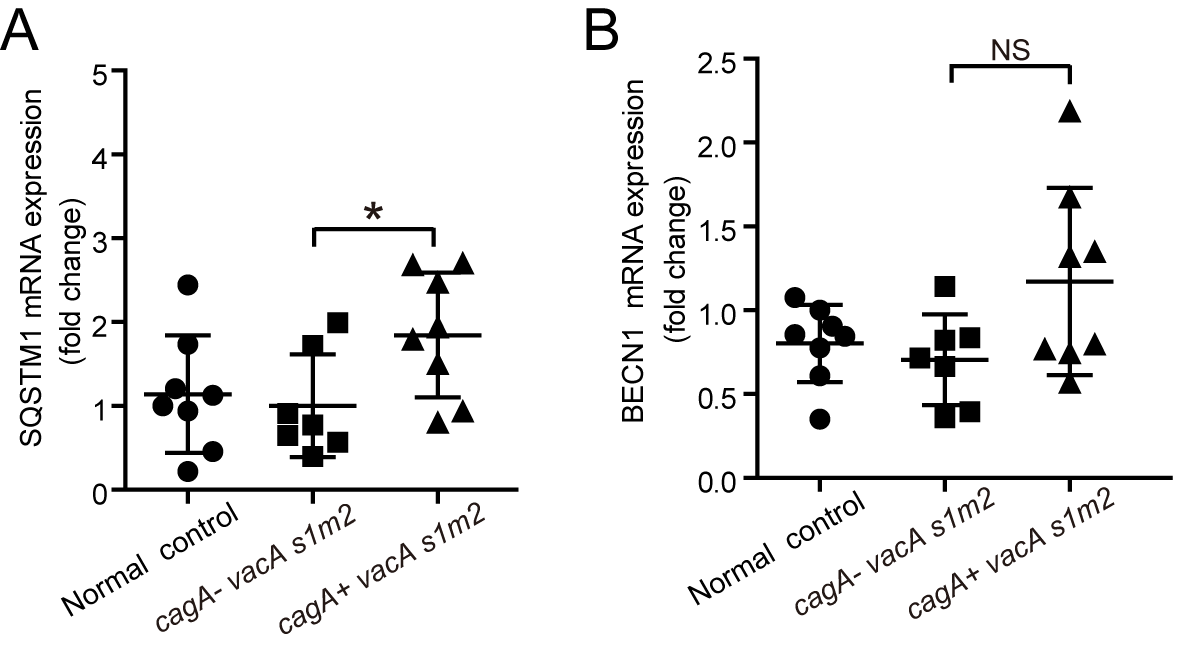


**Supplementary Figure 1** (**A** and **B**) Real-time PCR detection of SQSTM1 and BECN1 in gastric tissue infected with the indicated type of *H. pylori* strains. All real-time PCR data are normalized to β-actin and expressed as fold change. Data are representative of 6 independent experiments (* *P* < 0.05).

**
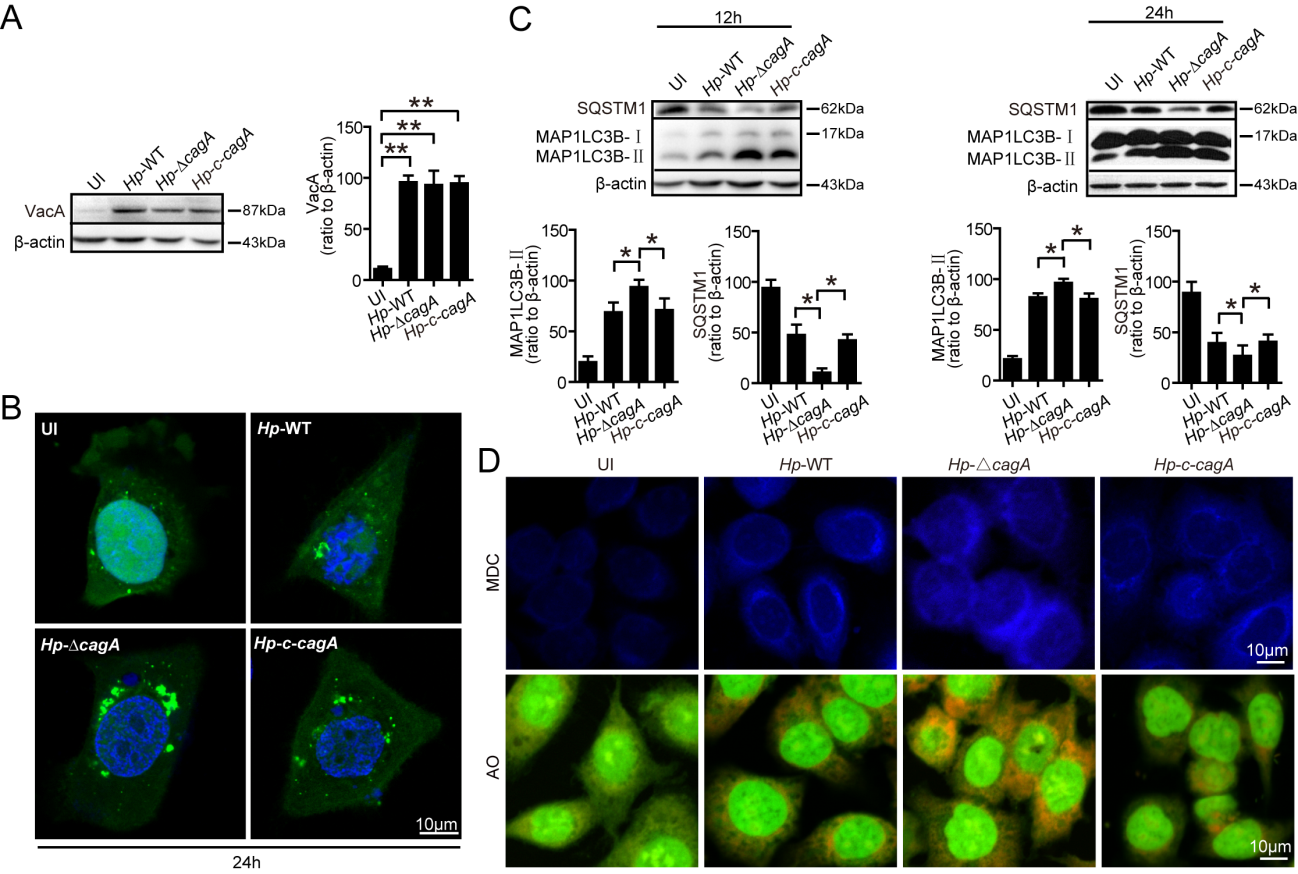
**

**Supplementary Figure 2** (**A**) Western blot analysis of VacA and β-actin in AGS cells after infection with *Hp*-WT, *Hp*-*∆cagA* or *Hp-c-cagA* (MOI=100:1) for 6 h. The intensity of VacA band was normalized to β-actin. (**B**) AGS cells were transfected with *GFP-MAP1LC3B*, and then infected with the indicated *H. pylori* strains for 24 h (MOI=100:1). Following fixation, cells were immediately visualized by confocal microscopy. The number of *GFP-MAP1LC3B* puncta in each cell (n≥200 cells) was counted. Scale bars: 10 μm. (**C**) AGS cells were treated with the indicated *H. pylori* strains (MOI = 100:1) for 12 or 24 h. The expression of MAP1LC3B-II conversion and SQSTM1 was detected by western blotting. (**D**) Representative images of MDC (upper) and AO (bottom) staining of AGS cells 6 h after infection with *Hp*-WT, *Hp*-*∆cagA* or *Hp*-*c-cagA* (MOI=100:1). For MDC staining (upper), punctate fluorescence in the cytoplasm indicates formation of autophagic vacuoles. For AO staining (bottom), red color intensity shows acidic vesicular organelles, representing autolysosomes. Scale bars: 10 μm. Results shown are representative of three independent experiments. **P*<0.05, ** *P*<0.01.

**
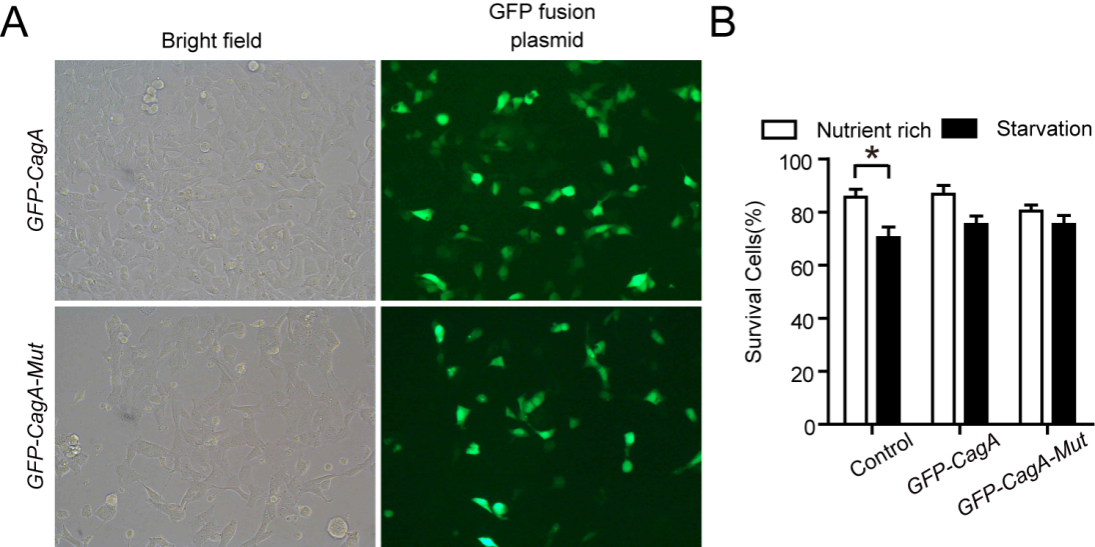
**

**Supplementary Figure 3** (**A**) AGS cells were transfected with 1000 ng of *GFP-cagA* or *GFP-cagA-Mut* plasmids for 24 h, fluorescence images of transfected cells were obtained. (**B**) AGS cells were transfected with either control or *cagA* expression plasmid for 24h before pretreatment of normal media or subjected to 4h starvation. Cell viability was assessed using an MTT assay. Results shown are representative of three independent experiments. **P*<0.05.

**
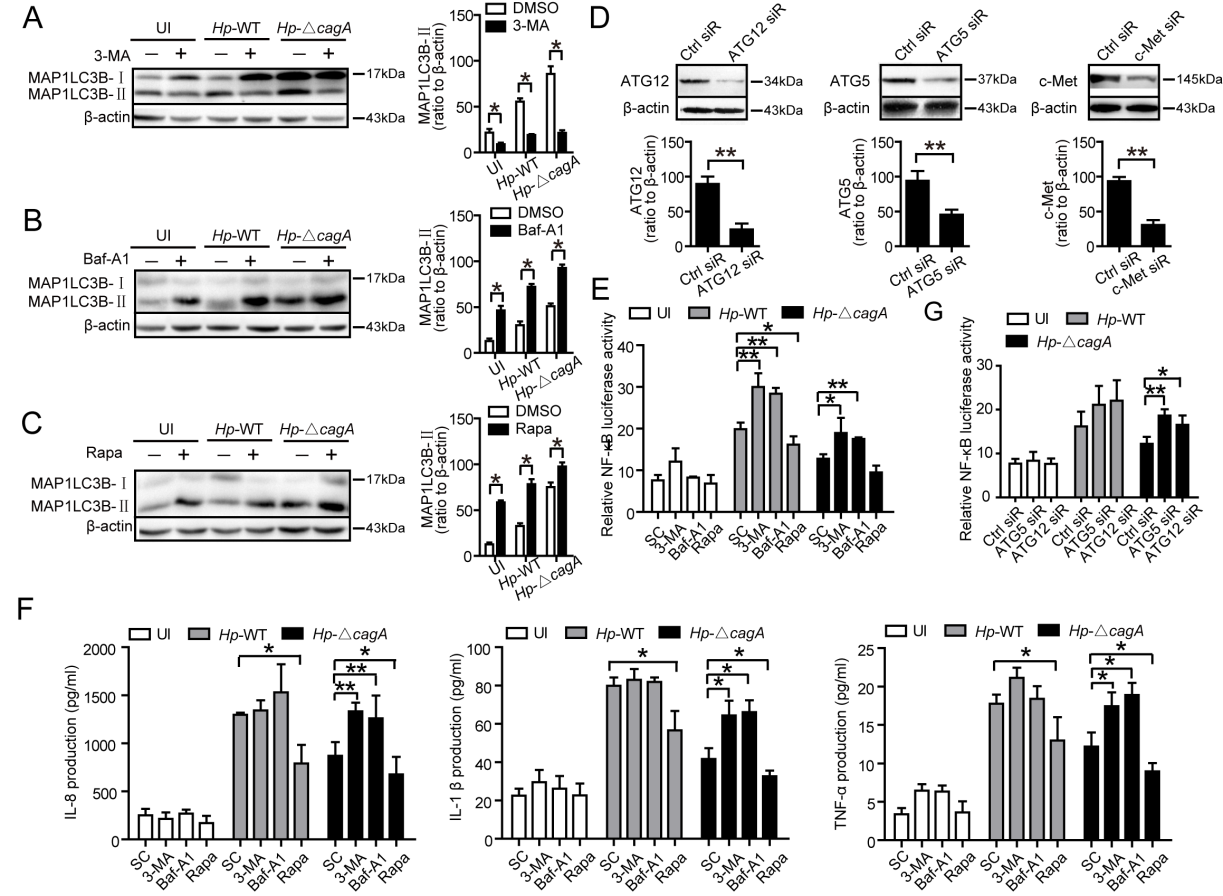
**

**Supplementary Figure 4** (**A**, **B** and **C**) AGS cells were infected with *Hp*-WT or *Hp-∆cagA* (MOI = 100:1) for 6 h in the presence of 2 mM 3-MA, 10 nM Baf-A1 or 100 nM rapamycin (Rapa). MAP1LC3B-II conversion was measured by western blot assay. (**D**) The inhibition efficiency of the siRNAs against ATG12, ATG5 or c-Met. AGS cells were transfected with siRNAs targeting ATG12, ATG5 or c-Met (100 nM each) for 24 h and the protein levels of the three targets were evaluated using western blot analysis. (**e**) (Left) After transfection with NF-κB-Luc for 24 h, AGS cells were infected with *Hp*-WT or *Hp-∆cagA* (MOI = 100:1) for 6 h pretreatment of the indicated inhibitors. Cells were analyzed by luciferase reporter assay. (Right) AGS cells were co-transfected with NF-κB-Luc and siRNA specific for ATG5 or ATG12 (50 nM) for 24 h and infected with *Hp-*WT or *Hp-∆cagA* (MOI = 100:1) for 6 h. Cells were analyzed by luciferase reporter assay. (**f**) After pretreatment of the indicated inhibitors, AGS cells were infected with *Hp-WT* or *Hp-∆cagA* (MOI = 100:1) for 24 h. Supernatants were assessed by ELISA for levels of IL-8, IL-1β and TNF-α. Data are presented as the means±SEM of three experiments. **P*<0.05, ** *P*<0.01.

**
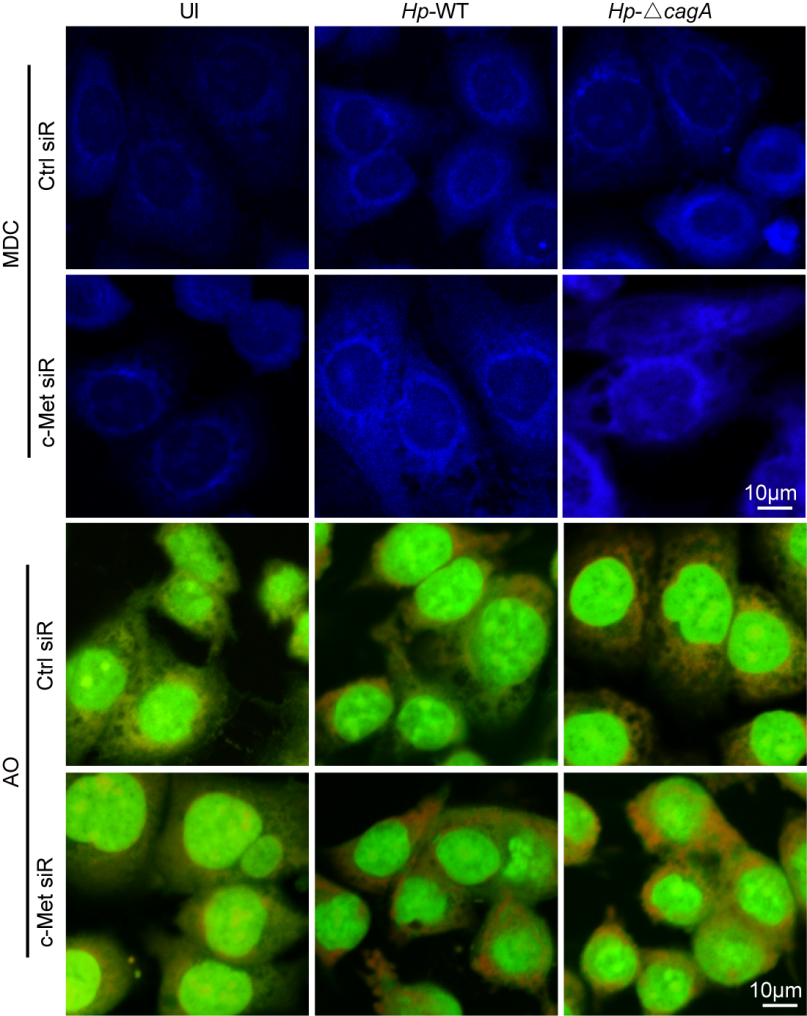
**

**Supplementary Figure** **5** (**A**) Representative images of MDC (upper) and AO (bottom) staining of AGS cells transfected with c-Met siRNA or control siRNA and then infected with *Hp*-WT or *Hp*-*∆cagA* for 6 h. Scale bars: 10 μm.

**Supplemental Table 1**

Sequences of primers used in the study

| Name | Sequences |
| --- | --- |
| *β-actin* | Forward: 5′-TTCCTTCCTGGGCATGGAGTCC-3′  Reverse: 5′-TGGCGTACAGGTCTTTGCGG-3′ |
| *IL-8* | Forward: 5′-GGCAGCCTTCCTGATTTCTG-3′  Reverse: 5′-GGGGTGGAAAGGTTTGGAGT-3′ |
| *TNF-α* | Forward: 5’-CCCAGGCAGTCAGATCATCTTC-3’  Reverse: 5’-GCTTGAGGGTTTGCTACAACATG-3’ |
| *IL-1β* | Forward: 5’-CATCAGCACCTCTCAAGCAG-3’  Reverse: 5’-ATAGCCGTACTCAAAAACCT-3’ |
| *BECN1* | Forward: 5'-CTGAGGGATGGAAGGGTC-3'  Reverse: 5'-TGGGCTGTGGTAAGTAATG-3' |
| *SQSTM1* | Forward: 5'-CTGCCCAGACTACGACTTGTGT-3'  Reverse: 5'- TCAACTTCAATGCCCAGAGG-3' |
| *CagA* | Forward: 5’-GAGTCATAATGGCATAGAACCTGAA-3’  Reverse: 5’-TTGTGCAAGAAATTCCATGAAA-3’ |
| *VacA-s* | Forward: 5’-ATGGAAATACAACAAACACAC-3’  Reverse: 5’-CTGCTTGAATGCGCCAAAC-3’ |
| *VacA-m* | Forward: 5’-CAATCTGTCCAATCAAGCGAG-3’  Reverse: 5’-GATAACAGCCAAGCTTTTGAGG-3’ |

**Supplemental Table 2**

Clinical characteristics of *H. pylori* positive and negative patients

| Characteristic | *H. pylori* positive  (n=106) | | | *H. pylori* negative  (n=11) |
| --- | --- | --- | --- | --- |
|  | *cagA* positive  (n=95) | *cagA* negative  (n=11) | *P* value |  |
| Age (median [range]) | 44 [23-57] | 42 [24-60] |  | 35 [26-55] |
| ≥50 | 27 | 4 | 0.584 |  |
| <50 | 68 | 7 |  |  |
| Sex |  |  |  |  |
| Male | 51 | 6 | 0.957 | 5 |
| Female | 44 | 5 |  | 6 |
| Smoker | 22 | 2 | 0.709 | 0 |
| Drinker | 11 | 1 | 0.805 | 0 |
| PPI consumption in previous days | 0 | 0 |  | 0 |
| NSAID consumption in previous days | 0 | 0 |  | 0 |
| Antibiotic consumption in previous days | 0 | 0 |  | 0 |
| *vacA* (s1/s2) | 95/0 | 11/0 |  |  |
| *vacA* (m1/m2) | 38/57 | 4/7 | 0.815 |  |
